# Comparison of internal jugular and subclavian access for central venous catheterization in pediatric cardiac surgery

**DOI:** 10.1186/cc10817

**Published:** 2012-03-20

**Authors:** A Pirat, A Camkiran, P Zeyneloglu, M Ozkan, E Akpek, G Arslan

**Affiliations:** 1 Baskent University, Ankara, Turkey; 2 Acibadem University, Istanbul, Turkey

## Introduction

Central venous catheterization (CVC) is an essential component of perioperative care in pediatric cardiac surgery. Traditionally the internal jugular vein (IJV) is used for CVC in cardiac surgery. The aim of this study was to compare IJV and subclavian vein (SV) routes for CVC in pediatric cardiac surgery in terms of success rate and mechanical and infectious complications.

## Methods

After Ethics Committee approval and written informed consent from the parents of the children were obtained, 200 children who were scheduled for cardiac surgery were randomly allocated to IJV (*n *= 100) and SV (*n *= 100) groups.

## Results

The mean age was 37 months (95% CI, 29 to 45 months) in group IJV and 35 months (95% CI, 29 to 42 months) in group SV (*P *= 0.619). The 95% CI for weight in groups IJV and SV were 10.4 to 14.2 kg and 10.2 to 13.0 kg, respectively (*P *= 0.595). The CVC success rates at first attempt for groups IJV and SV were 67% and 70%, respectively (*P *= 0.761). An alternative location was required to perform CVC in 90 patients in group IJV and in 92 patients in group SV (*P *= 0.806). The overall frequency of mechanical complications during the catheter insertion and its use was 26% in group IJV and 28% in group SV (log-rank test: *P *= 0.753). Significantly more arterial punctures occurred in group IJV than in group SV (14% vs. 4%, *P *= 0.024). Catheter tip misplacement was observed more frequently in group SV than group IJV (12% vs. 1%, *P *= 0.003). Catheter colonization rates were significantly higher in group IJV than group SV (15% vs. 5%, log-rank test: *P *= 0.020). There was no difference in bloodstream infection per 1,000 catheter days between group IJV and group SV (3.4 vs. 1.4, respectively: *P *= 0.319).

## Conclusion

In pediatric cardiac surgery patients, IJV and SV catheters had similar success rates as well as overall mechanical complication rates. Although the catheter colonization rate was significantly higher with IJV than SV, both access routes had similar rates of bloodstream infection.

